# Mediation of organismal aging and somatic proteostasis by the germline

**DOI:** 10.3389/fmolb.2015.00003

**Published:** 2015-01-23

**Authors:** Amirabbas Khodakarami, Isabel Saez, Johanna Mels, David Vilchez

**Affiliations:** Cologne Excellence Cluster for Cellular Stress Responses in Aging-Associated Diseases, University of CologneCologne, Germany

**Keywords:** aging, autophagy, Alzheimer's disease, germ cells, Huntington's disease, Parkinson's disease, proteasome, proteostasis

## Abstract

Experimental interventions that reduce reproduction cause an extension in lifespan. In invertebrates, such as *Caenorhabditis elegans*, the aging of the soma is regulated by signals from the germline. Indeed, ablation of germ cells significantly extends lifespan. Notably, germline-deficient animals exhibit heightened resistance to proteotoxic stress. This phenotype correlates with increased potential of intracellular clearance mechanisms such as the proteasome and autophagy in somatic tissues. Here we review the molecular mechanisms by which signals from the germline regulate lifespan in *C. elegans* with special emphasis on clearance mechanisms.

## Introduction

Fecundity and lifespan are negatively correlated, both under natural conditions or experimental interventions. Among invertebrates, birds and mammals, experimental paradigms that limit reproductive investment cause lifespan extension (Partridge et al., 2005). These findings suggest an evolutionary conserved pathway that links reproduction with longevity. However, the underlying mechanisms of this regulation are only beginning to be understood. Several hypotheses could explain the link between reproduction and lifespan. In one view, reproduction itself, or the processes enabling it, directly imposes somatic damage and reduces lifespan (Partridge et al., 2005). However, the disposable soma theory of ageing, formulated by Kirkwood (1977), has been supported by recent studies. Due to the limitation of nutrients in nature, organisms have to divide the available metabolic resources between reproduction and maintenance of the non-reproductive soma. Evolutionary pressure has been theorized to force a re-allocation of the resources in order to prevent, repair or eliminate damage to the germline, while little resources will be placed on the maintenance of somatic cells (Kirkwood, 1977). By this mechanism, the organism will ensure a healthy and fit progeny. Thus, somatic tissues undergo a progressive demise in their function and homeostasis. Conversely, it has been proposed that animals undergoing environmental stress such as a regimen of dietary restriction re-allocate their resources toward maintenance of somatic tissues, which results in lifespan extension and a delay in reproduction until more favorable survival conditions emerge (Shanley and Kirkwood, 2000). Notably, a recent study in *C. elegans* has shown that DNA damage in germ cells activates a systemic response that results in enhanced somatic tissue resistance through an increase in proteasome activity (Ermolaeva et al., 2013).

In support of the disposable soma theory of aging, limitation of reproduction by germ cells elimination in *C. elegans* and *D. melanogaster* provide effective mechanisms for extending lifespan (Hsin and Kenyon, 1999; Sgro and Partridge, 1999; Flatt et al., 2008), a phenotype that may be caused by heightened resource availability, increased autophagy and proteome stability within the post-mitotic soma (Lapierre et al., 2011; Vilchez et al., 2012b; Shemesh et al., 2013). Notably, castration has also been shown to increase lifespan in rodents and humans (Hamilton and Mestler, 1969; Drori and Folman, 1976; Min et al., 2012).

Here, we review recent insights into the regulation of longevity by the germline in *C. elegans*, with special emphasis on proteasome activity and autophagy.

### Signals from the germline regulate longevity

**Figure 1:**
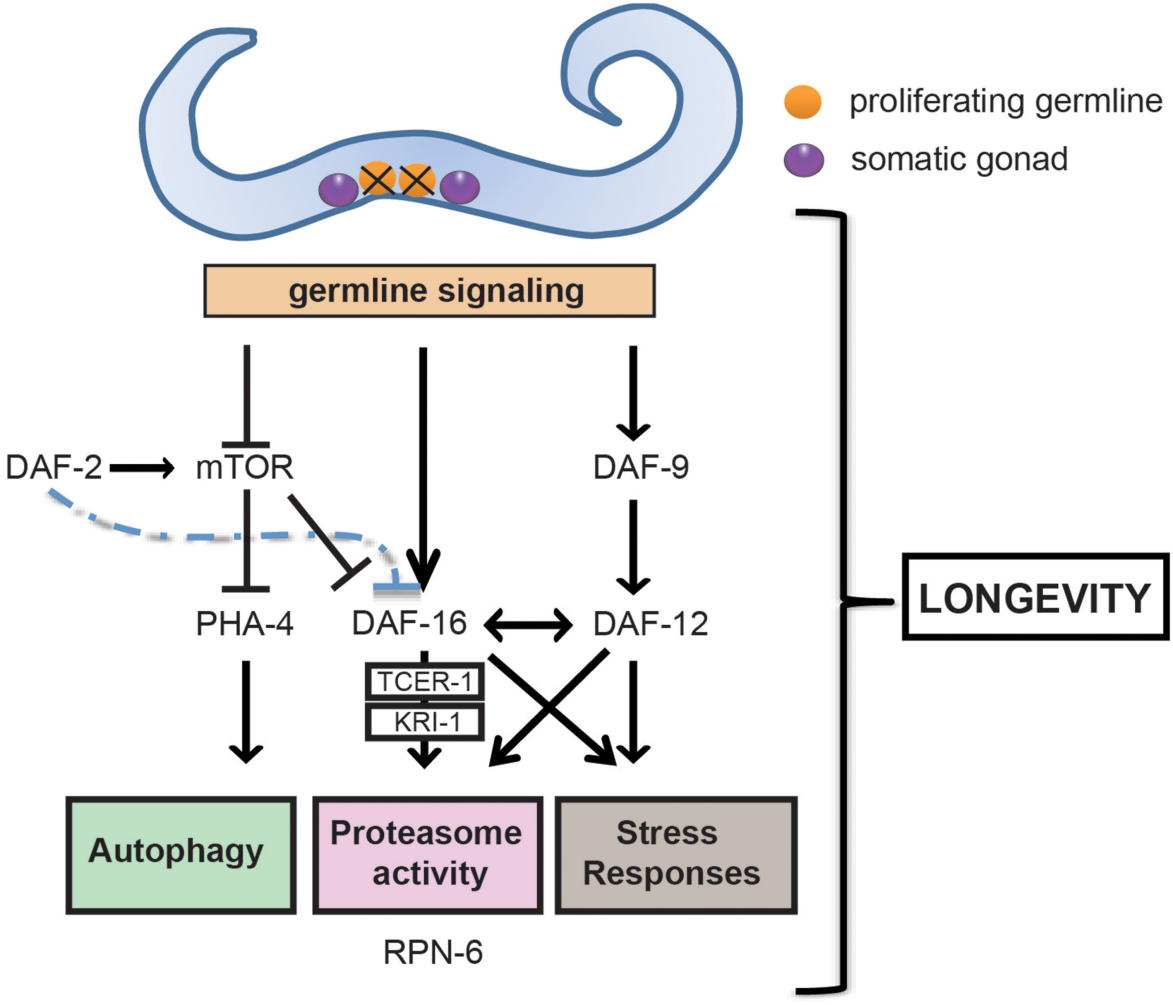
**Germline ablation modulates a series of pro-longevity transcription factors and clearance mechanisms that extends healthspan**. Removal of the germline, but not of somatic gonad, promotes healthspan and longevity in *C. elegans*. The ablation of the germline promotes longevity by triggering an active signaling network involving the nuclear localization and activation of FOXO transcription factor DAF-16. DAF-16 is negatively regulated by the *daf-2*/insulin/insulin-like growth factor (IGF) signaling (IIS) pathway and, when active, regulates downstream genes that activate the proteasome, increase stress resistance and ultimately extend lifespan. The induction of a number of these genes is dependent on TCER-1 and the ankyrin repeat- containing protein KRI-1. Germline loss also causes TOR (target of rapamycin) downregulation, which in turn stimulates *pha-4*, the worm FOXA forkhead transcription factor ortholog, and *daf-16* expression. Furthermore, activation through somatic gonad signaling of cytochrome P450 DAF-9, and of the steroid hormone receptor DAF-12 is necessary for the nuclear localization of DAF-16 and activation of stress resistance responses.

When proliferating germline cells of *C. elegans* are removed by either genetic or ablation interventions, worms live up to 60% longer than normal and are resistant to a variety of environmental stress conditions (Figure 1) (Hsin and Kenyon, 1999; Arantes-Oliveira et al., 2002; Wang et al., 2008). This extended longevity is not a result of sterility, because removing the entire reproductive system (the germline plus the somatic gonad) has no effect on lifespan (Hsin and Kenyon, 1999). In fact, this regulation is performed by the germline proliferating cells (Arantes-Oliveira et al., 2002), which are responsible for the generation of signals that modulate longevity (Wang et al., 2008).

In worms, the germline can be removed by laser microsurgery killing the two germline precursor cells. Germline ablation can also be reached by mutations in genes required for proliferation of germ cells (Kenyon, 2010). The most studied of these genes is *glp-1*, which encodes a N-glycosylated transmembrane protein homolog of Notch. *glp-1* is expressed in germline stem cells (GSCs) and promotes mitotic proliferation delaying the transition to meiosis (Austin and Kimble, 1987). Mutations in *glp-1* promote premature meiosis of germ cells resulting in long-lived germline-lacking adults (Arantes-Oliveira et al., 2002).

While ablation of the germline affords an efficient protection, the downstream effectors and complexity of such protection remain somewhat ambiguous. Germline removal promotes longevity by triggering an active signaling network, involving the nuclear localization and activation of DAF-16, a forkhead transcription factor (FOXO) (Panowski and Dillin, 2009). DAF-16 is the major downstream effector of the *daf-2/*insulin/insulin-like growth factor (IGF) signaling (IIS) pathway. The insulin/IGF-1 receptor activates a conserved PI3-kinase/PDK/AKT signaling cascade that phosphorylates FOXO transcription factors, thereby preventing its nuclear localization. When IIS signaling is reduced, FOXO translocates to the nucleus and regulates downstream genes that not only extend lifespan but also increase stress resistance (Figure 1) (Kenyon et al., 1993; Murakami and Johnson, 1996; Honda and Honda, 1999). Delayed aging, induced by IIS reduction, protects invertebrates and mice from protein aggregation toxicity (Morley et al., 2002; Kenyon, 2005; Cohen et al., 2006, 2009). Studies in *C. elegans* disease models have shown that expression of human disease proteins in invertebrates can be toxic and results in physiological and behavioral changes (Morley et al., 2002; Kerr et al., 2011). IIS reduction delays polyglutamine aggregation and toxicity, suggesting that this pathway modulates the protein homeostasis (proteostasis) network (Morley et al., 2002). In a *C. elegans* model of Alzheimer's disease, reduced function of the IIS can protect from the toxicity of Aβ_1−42_expression in a DAF-16 dependent manner (Cohen et al., 2006). Interestingly, reduced IIS induces the aggregation of small toxic Aβ_1−42_oligomers into larger, less toxic structures suggesting the activation of an aggregation mechanism (Cohen et al., 2006). However, it has been also suggested that autophagic degradation of the β-amyloid peptide is required for the protective effect of reduced IIS in Aβ_1−42_-expressing worms (Florez-McClure et al., 2007).

Similar to *daf-2* mutants, germline-lacking worms exhibit a *daf-16* dependent extension in lifespan. However, longevity caused by germline ablation functions in a synergistic manner with mutations in IIS receptor *daf-2* (Hsin and Kenyon, 1999). Notably, loss of germline further doubles the already-extended lifespan of IIS mutants (Hsin and Kenyon, 1999). Moreover, in germline-ablated worms but not *daf-2* mutants, activities of at least other three genes (*kri-1*, *daf-12*, and *daf-9*) are also required for the constitutive nuclear localization of DAF-16 (Figure 1) (Gerisch et al., 2001; Berman and Kenyon, 2006). *kri-1* encodes an intestinal ankyrin repeat protein which is orthologous to the human disease gene KRIT1/CCM1 (Krev interaction trapped/cerebral cavernous malformation 1) (Sahoo et al., 1999). *daf-12* encodes a steroid hormone receptor which is homologous to human vitamin D receptor. *daf-9*, a cytochrome P450, is thought to make or modify a lipophilic ligand for *daf-12*. In addition, the transcription elongation factor, TCER-1, is necessary for the increased expression of several DAF-16-target genes in germline-lacking worms but it is dispensable for up-regulation of DAF-16-target genes in IIS mutants (Ghazi et al., 2009). TCER-1 levels increase in the intestine in response to germline ablation. This increase is sufficient to trigger key downstream pathways, since ectopic expression of *tcer-1* extends the lifespan of wild-type animals with an intact reproductive system. Gonadal signaling also modulates DAF-16 activity in a tissue specific manner and stage different from IIS. Whereas IIS reduction causes nuclear localization of DAF-16 in most cell types, DAF-16 localizes mostly in the nucleus of intestinal cells during the first day of adulthood in germline-lacking worms (Antebi, 2013). The intestine of *C. elegans* stores fat, produces yolk, secretes insulin like-peptides, and thus acts as the entire endoderm. It is also central for the gonadal longevity, as expression of *daf-16* specifically in the intestine completely restores the lifespan extension of germline-defective *daf-16* mutant animals (Kenyon, 2010).

In addition to *daf-16*, loss of germline-mediated longevity requires other transcription factors: *hsf-1, skn-1*, and *pha-4* (Hsu et al., 2003; Lapierre et al., 2011; Vilchez et al., 2012b). *hsf-1* is necessary for the regulation of heat-shock response and adult lifespan (Hsu et al., 2003). *skn-1* is the worm ortholog of *nrf-2* and plays a key role in oxidative stress response (Saez and Vilchez, 2014). Loss of germline induces TOR (target of rapamycin) downregulation, which in turn stimulates *pha-4* (Lapierre et al., 2013), the worm FOXA forkhead transcription factor ortholog. It is important to remark that both *skn-1* and *pha-4* are not only required for the longevity phenotype induced by germline-loss (Lapierre et al., 2011; Vilchez et al., 2012b) but also dietary restriction (Bishop and Guarente, 2007; Panowski et al., 2007). It has been also reported that *nhr-80*, a nuclear hormone receptor, links fatty acid desaturation to lifespan extension through germline removal in a *daf-16* independent manner (Goudeau et al., 2011).

The *glp-1* longevity is to some extent also regulated by microRNAs (miRNAs). Loss of *mir-71* completely abrogates the germline precursor ablation (Boulias and Horvitz, 2012). Strikingly, neural expression of *mir-71* is enough to rescue the gonadal longevity, demonstrating a cell-non-autonomous relationship between the gonad, intestine and the nervous system. Gonadal signals that activate DAF-12 also activate its miRNA targets, *mir-84* and *mir-241* (Antebi, 2013). These miRNAs down-regulate *lin-14, akt-1*, and possibly other targets, which stimulate DAF-16 transcriptional activity and, thus, extending lifespan. Because deletion of miRNA does not totally abolish DAF-16 activity, other signals from the gonad could also induce the longevity phenotype (Antebi, 2013).

### Cellular mechanisms of protein degradation

The proteome of the cell is under constant challenge during the aging process. Proteostasis is maintained by a complex network of quality control mechanisms that monitor synthesis, folding, concentration, cellular localization, interactions and ultimately degradation of proteins in the cell (Powers et al., 2009). The two main cellular proteoytic systems are the ubiquitin proteasome system (UPS) and autophagy. The UPS is the primary selective mechanism of protein degradation in eukaryotic cells (Schmidt and Finley, 2014). The UPS is a carefully timed and precise mechanism which is critical for maintaining the appropriate levels of many regulatory proteins involved in several pathways such as signal transduction, metabolism or cell cycle (Finley, 2009; Wong and Cuervo, 2010; Buckley et al., 2012; Okita and Nakayama, 2012; Vilchez et al., 2012a; Tanaka and Matsuda, 2014). The UPS is not only necessary to degrade regulatory proteins but it is also an essential component of the proteostasis network necessary for eliminating damaged, misfolded and aggregation-prone proteins (Finley, 2009; Wong and Cuervo, 2010; Tanaka, 2013). The first step of the UPS-mediated proteolysis is the conjugation of ubiquitin through a sequential mechanism that targets proteins for degradation. The polyubiquitylated proteins are then recognized, unfolded and finally cleaved into small peptides by the proteasome (see Vilchez et al., 2014a for detailed review).

The proteasome is a complex proteolytic machine formed by the assembly of several subunits (Coux et al., 1996). The core particle (20S) of the proteasome consists of 28 subunits, which are assembled into four seven-membered rings and exhibit a barrel-like structure (Coux et al., 1996). Although 20S particles can exist in a free form, they are considered to be inactive due to its closed form and binding to proteasome activators is required for degradation of polyubiquitylated proteins (Kisselev and Goldberg, 2005). However, free 20S particles can degrade small proteins in an ATP- and ubiquitination-independent manner (Baugh et al., 2009).

The most common active proteasome results from the assembly of the 20S and the 19S (26S, single capped or 30S, double capped) (Finley, 2009). The 19S regulates the activity of the complex and is responsible for recognizing, unfolding and translocating polyubiquitylated proteins to the 20S for degradation in an ATP dependent manner (Finley, 2009; Tanaka and Matsuda, 2014). Notably, proteins and even protein aggregates can also be degraded in an ubiquitin-independent way by free 20S or by PA28-binding 20S particles (Dubiel et al., 1992; Ma et al., 1992; Garcia-Mata et al., 1999; Baugh et al., 2009).

Parallel to the proteasome, autophagy is the main cellular clearance pathway (see Vilchez et al., 2014a for detailed review). Cytosolic fractions, organelles and macromolecules are degraded by autophagy through the lysosome. In energy-demanding situations, such as nutrient-deprivation, autophagy degrades many different substrates to fulfill the energetic requirements of the cell (Egan et al., 2011; Rubinsztein et al., 2011). These substrates include macromolecules that provide energy and nutrients during starving periods (Ravikumar et al., 2010). Moreover, due to its capacity to engulf whole cellular regions, autophagy is essential in processes that require extensive cellular restructuration, such as embryogenesis, cellular differentiation or cellular death (Cuervo, 2004; Mizushima et al., 2008). The catalytic components of autophagy are the lysosomes. Lysosomes contain a large variety of cellular hydrolases, proteases, lipases, nucleotidases and glycosidases that show highest activity at acidic pH. Autophagy is required for maintaining cellular homeostasis, acting as a quality control mechanism of proteins and organelles. Thus, autophagy can degrade aggregates of neurodegenerative-associated proteins such as tau, α-synuclein and polyglutamine-expanded proteins (Martinez-Vicente and Cuervo, 2007). Lysosomal proteolysis results in small di and tri-peptides and free aminoacids that are released into the cytosol to be further metabolized to obtain energy or recycled to synthesize the novo proteins (Ravikumar et al., 2010; Mizushima and Komatsu, 2011). Although initially considered a non-selective mechanism, molecular chaperones and other cargo-recognition molecules have been shown to mediate the degradation of specific proteins through the lysosome (Wong and Cuervo, 2010).

### Differences between somatic and germline proteostasis with age

Loss of proteostasis is considered one of the hallmarks of aging (Lopez-Otin et al., 2013) and contributes to multiple age-related diseases such as Alzheimer's (Selkoe, 2011), Parkinson's (Bosco et al., 2011) or Huntington's disease (Finkbeiner, 2011). Somatic and germline tissues exhibit different activation and maintenance of proteostasis mechanisms (Fredriksson et al., 2012; Tsakiri et al., 2013). These findings raise an intriguing question: How is the proteostasis network affected in different cell types during aging? The specific biological purpose of every cell type may define cellular differences in proteostasis and how aging impacts on their protein clearance machinery (Vilchez et al., 2014a). Recent findings in cells that do not age such as embryonic stem cells (ESC) support this hypothesis. Accordingly, ESCs exhibit high proteasome activity compared with their differentiated counterparts (Vilchez et al., 2012a). This increased proteasomal activity might be required for ESCs to maintain an intact proteome for self-renewal and avoid senescence. Interestingly, oxidized proteins are removed during differentiation of ESCs in a proteasome-dependent manner (Hernebring et al., 2006), suggesting that the differentiation process itself also has an influence on protein clearance mechanisms.

In adult organisms, GSCs maintain an unlimited proliferative capacity to fulfill their biological purpose: to be passed from one generation to the next. GSCs can acquire *in vitro* properties similar to those of ESCs such as pluripotency (Guan et al., 2006). GSCs are the origin of the gametes that will generate the embryos. ESCs and oocytes share a common transcriptome signature (Assou et al., 2009). Similar to hESC, human oocytes show an increased expression of specific proteasome subunits (Assou et al., 2009). In *D. melanogaster*, gonads (ovaries and spermathecae) and maturating oocytes have an increased 26S proteasome activity and accumulate less damaged proteins compared to aging somatic tissues. Proteasome activity is already down-regulated in middle-aged flies, when signs of aging first appear (Fredriksson et al., 2012; Tsakiri et al., 2013). In contrast to their age-matched somatic tissues, maturating oocytes and gonads maintain their enhanced proteasome activity during the aging process (Fredriksson et al., 2012). However, *C. elegans* germline cells show enhanced levels of oxidized proteins, which are removed by the proteasome during oocyte maturation, suggesting that the high proteasome activity might, in turn, be regulated during differentiation in order to eliminate damaged proteins (Goudeau and Aguilaniu, 2010). Thus, more studies are needed to shed light into the regulation of damaged proteins in ESCs and germline cells and the changes triggered during differentiation.

Increased proteasome activity in maturating oocytes and gonads may contribute to ensure the generation of an intact proteome in the following generation. In line with the disposable soma theory of aging, these enhanced proteostasis mechanisms in the germline would allow organisms to avoid replicative senescence by establishing an aging (somatic tissues) and rejuvenated/immortal (germ cells) lineage.

Importantly, post-mitotic somatic cells hold an especial distinction for their susceptibility to age-onset protein aggregation disorders. As the somatic cell ages, the accumulation of misfolded proteins represents a challenge to the aging cell, especially as they aggregate in inclusions capable of overwhelming the cellular machinery required for their proteolysis. This is the case for polyglutamine, β- amyloid and α-synuclein aggregates, which inhibit the proteasome (Gregori et al., 1995; Bence et al., 2001; Bennett et al., 2005; Tseng et al., 2008; Zhang et al., 2008). However, there is some controversy since some studies report proteasome activity to be unaffected by polyglutamine inclusions (Bowman et al., 2005). The effect of ageing on misfolded proteins might be also a result of age-related dysregulation of chaperones, downregulation of degradation machinery itself, and a progressive accelerating loss in cellular homeostasis. As such, a rapid decline in the ability of the cell to protect its proteome has been correlated with several age-related disorders (Balch et al., 2008; Powers et al., 2009). This conversely suggests that the long-lived somatic cells, such as those found in a germline-lacking animals, could exhibit a heightened capacity for clearing damaged toxic proteins, and that this proteostatic potential might contribute to the extended longevity in these animals.

### Modulation of somatic proteostasis by the germline

In worms, the ability to maintain proteostasis dramatically decreases in somatic tissues once reproduction starts. Notably, mutations that induce a germline arrest delay the demise in proteostasis of somatic cells (Shemesh et al., 2013). As a result, germline-lacking worms are more resistant to proteotoxic conditions such as heat stress. These nematodes are also more protected from protein aggregation and polyglutamine toxicity. Modulation of somatic proteostasis by the germline depends on the factors also required for the longevity phenotype, including *daf-16, tcer-1, kri-1*, *daf-12*, *daf-9*, *nhr-80*, *pha-4*, and *hsf-1* (Vilchez et al., 2012b; Shemesh et al., 2013). Enhanced proteostasis maintenance in somatic tissues induced by lack of germline could be explained by increased activity of the two main intracellular protein clearance mechanisms: the proteasome and the autophagy-lysosome systems. Interestingly, germline-lacking worms exhibit increased proteasome activity in their somatic tissues (Vilchez et al., 2012b). These enhanced proteasome activity is induced by up-regulated expression of the 19S proteasome subunit *rpn-6*, the worm ortholog of *PSMD11*. RPN-6/PSMD11 stabilizes the interaction between the 19S cap and the 20S and is key for the activity of the proteasome (Pathare et al., 2012; Vilchez et al., 2012a). Moreover, its ectopic expression is sufficient to increase proteasome activity and protect from the accumulation of toxic protein aggregates in Huntington's disease models (Vilchez et al., 2012a,b). In these long-lived animals, DAF-16 regulates increased proteasome activity, *rpn-6* expression and longevity (Vilchez et al., 2012b). Similarly, one of the orthologs of *daf-16*, FOXO4, is necessary for increased proteasome activity and RPN-6/PSMD11 levels in immortal hESCs (Vilchez et al., 2012a, 2014b). These results suggest that DAF-16/FOXO crosses evolutionary boundaries and links ESC function to invertebrate longevity regulation.

Notably, germline-lacking worms have increased autophagy activity. In these long-lived worms, autophagy and lipase-4-dependent lipolysis have been shown to be interconnected and modulate the longevity phenotype (Lapierre et al., 2011), which could be caused by an increased lipid clearance or regulation of signaling compounds derived from lipids (Folick et al., 2015). mTOR and the PHA-4 transcription factor mediate this response by modulating the expression of several autophagy genes. Another transcription factor, HLH-30/TFEB, also activates autophagy in the germline-lacking worms. However, HLH-30/TFEB may be an universal regulator of longevity since it is required not only for the lifespan of germ-less worms but also other long-lived mutants (Lapierre et al., 2013).

While autophagy is required for the longevity phenotype of germline-lacking worms (Lapierre et al., 2011), the nature of the degraded molecules remains unknown. Thus, it will be fascinating to determine whether autophagy-dependent degradation of proteins rather than of other components is required for the lifespan extension in germline-lacking worms. Furthermore, the role of cargo-recognition molecules in this prolongevity mechanism could contribute to understand the molecular basis of the aforementioned diseases.

In addition to increased proteasome activity and autophagy, enhanced stress resistance and an increase in chaperone levels could also mediate the improvement in somatic proteostasis (Wong and Cuervo, 2010; Tanaka and Matsuda, 2014).

## Concluding remarks

Longevity-promoting pathways increase proteostasis mechanisms, a factor that contributes to ameliorate age-related diseases. The studies in invertebrates provide new insights into the molecular mechanisms of these diseases and could facilitate further experiments in higher organisms. This could finally be translated into novel approaches for the treatment of progressive, age-related neurodegenerative diseases. Removing the germline of *C. elegans* has been proved as an effective mechanism to extend lifespan, maintain proteostasis and protect from the accumulation of toxic protein aggregates associated with diseases such as Huntington's disease. This regulation seems to be achieved by conferring germ-like proteostasis features to the somatic tissues. Further understanding of this regulation and the proteostasis nodes activated by loss of germline may reveal innovative therapeutic approaches for the treatment of age-related diseases.

### Conflict of interest statement

The authors declare that the research was conducted in the absence of any commercial or financial relationships that could be construed as a potential conflict of interest.
